# The Effect of Scanning Strategy on the Thermal Behavior and Residual Stress Distribution of Damping Alloys during Selective Laser Melting

**DOI:** 10.3390/ma17122912

**Published:** 2024-06-14

**Authors:** Zhiqiang Yan, Kaiwen Wu, Zhongmin Xiao, Jizhuang Hui, Jingxiang Lv

**Affiliations:** 1 School of Mechanical and Aerospace Engineering, Nanyang Technological University, Singapore 639798, Singapore; yzq18829548460@163.com (Z.Y.); kwu010@e.ntu.edu.sg (K.W.); 2 Key Laboratory of Road Construction Technology and Equipment of MOE, Chang’an University, Xi’an 710064, China; huijz@chd.edu.cn (J.H.); lvjx@chd.edu.cn (J.L.)

**Keywords:** damping alloy, selective laser melting, numerical modeling, scanning strategy, thermal behavior, residual stress

## Abstract

The manufacture of damping alloy parts with stable damping properties and high mechanical performances in the selective laser melting (SLM) process is influenced by temperature evolution and residual stress distribution. Choosing an appropriate scanning strategy, namely the specific trajectory along which the laser head scans powders within given area, is crucial, but clearly defined criteria for scanning strategy design are lacking. In this study, a three-dimensional finite element model (FEM) of the SLM process for manufacturing a WE43 alloy component was established and validated against the published experimental data. Eleven different scanning strategies were designed and simulated, considering variables such as scanning track length, direction, Out–In or In–Out strategy, start point, and interlayer variation. The results showed that scanning strategy, geometry, and layer number collectively affect temperature, melt pool, and stress outputs. For instance, starting scanning at a colder part of the powder layer could lead to a high peak temperature and low melt pool depth. A higher layer number generally results in lower cooling rate, a lower temperature gradient, a longer melt pool life, and larger melt pool dimensions. Changing the start point between scanning circulations helps mitigate detrimental residual stress. This work highlights the potential of analyzing various scanning strategy-related variables, which contributes to reducing trial-and-error tests and selecting optimal scanning strategies under different product quality requirements. This article can assist in the design of appropriate scanning strategies to prevent defects such as element loss due to evaporation, poor bonding, and deformation or cracking from high residual stress. Additionally, identifying stress concentration locations and understanding the effects of geometry and layer number on thermal and mechanical behaviors can assist in geometry design.

## 1. Introduction

Damping alloys, as one of the most commonly used functional alloys (e.g., for automobile [1], construction [2], and aviation [3]), have the ability to suppress unnecessary noise and vibration during engineering applications [4]. This is due to its ability to convert the mechanical energy of vibration into heat that can be dissipated, a phenomenon called intrinsic mechanical damping or internal friction [5]. Common damping alloys include aluminum alloys [6], magnesium alloys [7], and Mn-Cu-based alloys [8]. Their damping properties are based on different mechanisms and can be divided into four categories [9]: complex, ferromagnetic, dislocation, or twinned damping alloys. Many engineering applications involve extreme environmental conditions, limiting the choice of damping alloys. Ferromagnetic and twinned damping alloys might not be applicable as they would lose their damping properties at high temperatures or under magnetic fields, while complex and dislocation damping alloys retain their damping properties under these situations [5]. Magnesium alloys, as a type of dislocation alloy, are widely used in aerospace and biomaterial applications due to their advantages like low density, a high specific strength, and stable damping properties [10]. Since pure magnesium has poor high-temperature strength and corrosion resistance, the addition of alloying elements (Al, Mn, Zn, Si, etc.) has proven to be an effective way to improve it. In recent years, the addition of rare earth elements (RE) has aroused the interest of researchers. Mg-RE alloys like Mg-Gd, Mg-Y, Mg-Nd, and Mg-Sm could obtain stronger mechanical properties and a higher relative density compared to traditional magnesium alloys. WE43, a type of Mg-Y-based alloy, has been widely used in aerospace applications. An effective damper structure on electronic packages for spacecraft designed by P. Veeramuthuvel [11] could be manufactured with this kind of material to enhance its damping capability.

Conventionally, magnesium alloy parts are manufactured through methods like deformation processing, casting, and powder metallurgy (P/M) techniques. However, these methods have their limitations. Deformation processing, due to the hexagonal closed packing (HCP) structure of magnesium, needs to be performed at elevated forming temperatures, which might result in poor surface quality, oxidation, and low efficiency. Casting, currently the most commonly used method, can ensure high efficiency and precision, but it is difficult to create near-net-shape structures with complex shapes and internal features. Additionally, the strong oxidizing tendency of magnesium can degrade product quality due to the thermodynamically stable phases formed during solidification [12]. With the development of additive manufacturing (AM), new powder-based manufacturing methods offer the possibility of making high-quality and near-net-shape magnesium alloy [13], as well as magnesium matrix composite [14] products with relatively high efficiency. Selective laser melting (SLM), one of the widely used 3D printing methods, has been established as an effective way to produce metal parts. It offers advantages such as high dimensional precision and good surface integrity without the need for subsequent processing (near-net shape), as well as design freedom, reduced material wastage, and the elimination of expensive tooling [13]. The SLM process involves several steps [15]: first, the CAD data (in STL format) must be preprocessed by software such as Magics 28 to generate supports for overhanging features and slice data for the laser scanning of individual layers. Next, a thin layer of metallic powders is laid on the substrate plate (usually of the same material as the powders, but dissimilar materials could also be applied [16]) within the building chamber (typically filled with inert gas to prevent oxidation). After that, a high-power density laser melts and fuses the metallic powders within selected areas based on the slice data. Once the laser scanning is complete, the platform is lowered, a new layer of metallic powders is deposited on top of the current layer, and the laser scanning begins again. Parameters like laser power, scanning speed, hatch spacing, and layer thickness may be adjusted to achieve optimal fusion performance. This process is repeated many times until the part is completely produced. Finally, loose powders are removed from the building chamber, and the part is separated from the substrate plate manually or through electrical discharge machining (EDM). Additionally, some SLM machines can pre-heat the substrate plate or the building chamber, which helps to reduce the residual stresses caused by the inherently large thermal gradients caused by the high heating and cooling rates during manufacturing, thereby improving the final part’s quality [17]. The type of laser wave (continuous or pulsed) can influence the mechanical behavior [18]. Other AM techniques, such as wire and arc additive manufacturing (WAAM), are also applicable to magnesium alloy manufacturing [19]. Moreover, numerous studies have revealed that SLM is capable of producing complex, dense damping alloys (e.g., Ni-Ti, which is commonly used for shape memory alloy parts [20], whose microstructure and Elinvar effect have been investigated [21], and medical implants [22]; other alloys include Ti6Al4V [23], 316L [24], Mn-xCu [25], etc.), especially Mg-RE alloys (like WE43 [26]), with better mechanical and damping properties. This offers a promising approach to extending the service lives of certain engineering structures. For instance, applying an SLMed damping alloy in the repair of a pressure vessel–reactor mantle could have a positive effect, enhancing its impact strength [27]. However, during the SLM process, the inappropriate selection of process parameters like scanning speed and power may lead to defects such as porosity, poor dimensional accuracy, and element loss caused by evaporation [28]. Additionally, metal powders undergo a complex heat history involving a complex thermal cycle and phase transformation within a short period during SLM, which can result in an uneven temperature distribution and sharp temperature gradients that make the formed parts prone to stress-cracking, warpage, and deformation [29]. Therefore, it is crucial to find a way to precisely predict the temperature field and stress field during SLM.

Fortunately, numerical models (considering a heat transfer model that can provide temperature results [30] and a mechanical model that can provide stress results [31]) have been developed to predict temperature and stress distribution, as well as the thermos-fluid dynamics of the melt pool during the SLM process [32], using finite element analysis (FEA) software like Mechanical APDL (Ansys Parametric Design Language) 2023 R1. Moreover, numerical simulation can help in understanding other AM processes [33] like field-assisted additive manufacturing (FAAM) [34]. According to [29], the three-dimensional transient temperature field of a damping alloy during the selective laser melting process can be simulated by Mechanical APDL 2023 R1 considering its thermophysical properties (i.e., thermal conductivity, specific heat, and density as functions of temperature) and the phase transformation latent heat of the material. The simulation program can therefore be used to investigate how, under different conditions, the temperature distribution, temperature gradient, and cooling rate of the damping alloys vary during SLM. Regarding mechanical behavior, under the laser heat source, the expansion or contraction of the material is constrained by the surrounding material to generate stress. Therefore, the stress field of the SLM is simplified as a thermo-elastic-plastic problem in the heating and melting zone [35]. The numerical simulation of residual stress within the SLMed part is based on an elastoplastic constitutive law, which predicts the residual stress using the strains induced by the thermal gradients [31]. The accuracy of these models has been verified for several different materials. Yali Li and Dongdong Gu [36] investigated the effects of scanning speed and laser power on SLM thermal behaviors of commercially pure titanium powder via numerical simulation and verified them with the experimental results; Zhibo Luo and Yaoyao Zhao [37] conducted similar research on the temperature field of stainless steel 316L; Zhanyong Zhao et al. [38] researched the 7075 Al alloy through simulation and experiment. Regarding SLMed damping alloys, Chenyu Su et al. [29] studied the thermal behaviors of two typical damping alloys, AZ31 Mg and Mn-Cu alloy, during SLM, while Wenli Wang et al. investigated both the thermal [39] and mechanical [35] behaviors of a typical Mg-RE alloy: the Mg-Y-Sm-Zn-Zr alloy. In addition to overall temperature and stress fields, research on the development of melt pools has also been conducted via the thermo-fluid-dynamic modeling of the AZ91D Mg alloy [40]. Another important factor that could influence the temperature and stress fields during SLM is the scanning strategy: how the laser head scans the powders within each layer. Its influence has also been studied by previous researchers: Pramod R. Zagade et al. [41] built an analytical model to reveal the effect of scanning strategy on the temperature field and melt track dimensions; Peiying Bian et al., as well as Zhijun Zheng et al., conducted simulations and experiments to investigate how the scanning strategy influences the residual stress [42] and manufacturing quality [43] of 316L steel; Jinbiao Zhou et al. [44] studied the scanning strategy’s effect on the temperature history and residual stress of SLMed Ti-6Al-4V components; Xiaochuan Zhang et al. [45] researched its effect on the stress and deformation of overhang structures. Various scanning strategies have been proposed. For a plate, examples include stripe and chessboard (island) scanning strategies [42], unidirectional, bidirectional, and Out–In and In–Out strategies. [41] Moreover, the scanning strategy not only could vary within the same layer between different sets but also could vary between different layers within a single set, including different trajectories and starting points [44]. Their research revealed a close connection between residual stress and scanning strategy. Important factors that influence residual stress include scanning track length, Out–In or In–Out strategy, direction, start point, depth into the surface, etc. In addition to adjusting the scanning strategy, some post-processing measures like shot peening, thermal stress relief, or vibratory stress relief [46] can also help to uniformly relieve the residual stresses. Moreover, in terms of bio-application, the residual stress induced by an ultrashort pulsed laser (USPL) on a monocrystalline silicon substrate can be used as a stimuli for cell modulation, while the magnitude of residual stress can be minimized by adopting a lower repetition rate of the laser [47], showing the potential of the application and minimization of laser-induced residual stress from a microscopic perspective.

All in all, a damper structure on electronic packages made with damping alloy WE43 could be manufactured via selective laser melting. However, the uneven temperature distribution and rapid cooling that occur during the SLM process could cause high residual stress and problems like cracking, warpage, and deformation. A too-high or too-low temperature would also lead to defects. Among many SLM factors, the scanning strategy could have a great effect on residual stress and final quality. Thus, it is necessary to find the relationship between the temperature evolution mechanism, residual stress distribution, and scanning strategy-related variables.

In this work, the thermal–structural coupling finite element model of the WE43 alloy formed by SLM was established using Ansys Parametric Design Language (APDL) coding. Firstly, the thermophysical properties of WE43 and SLM process parameters were input into the program. Then, the simulated geometry of the damper structure was created and meshed. After that, a Gaussian heat source was created and moved along pre-designed scanning strategies, layer by layer. The FEA software conducted temperature field calculations, followed by stress field calculations. Finally, outputs regarding temperature, melt pool, and stress were gathered and compared, and the characteristics of different scanning strategies were generalized.

The contribution of this work can be summarized as follows, with an investigation into the current research and unresearched areas in this field: (a) Numerical models for thermal and mechanical behaviors during the SLM process have been well established and verified, but until now, most research was conducted based on SLMed block geometry, with scanning strategies designed accordingly for a plate layer [41]. However, SLM is usually used to manufacture more complex geometries, which require completely different scanning strategies. In this work, a supporting structure for electronic packages [11] was designed and simulated, and various scanning strategies were correspondingly developed. Moreover, this structure was designed to function as a particle damper; manufacturing it with the damping alloy WE43 could enhance its damping performance. Therefore, this article demonstrates greater practical engineering significance by revealing the relationship between scanning strategy and temperature and stress fields, enabling the design and optimization of scanning strategies in the SLM process for producing more complex geometries. (b) Several scanning strategy analyses have been conducted for different materials, e.g., 316L [42] and Ti-6Al-4V [44]. However, such research on Mg-RE alloy is seldom seen. Rongrong Xu et al. [35] conducted similar research on a Mg-Y-Sm-Zn-Zr alloy, but for WE43; only some previous research concerning WE43 products’ melt pool dynamics [48], macro-/microstructure [49], and mechanical testing results [50] has been published. In this article, the scanning strategy analysis of WE43 deepens our understanding of the material’s characteristics. (c) Previously designed scanning strategies by other researchers are usually arbitrary, and criteria indicating the effects of some common scanning strategy-related variables are lacking. In this article, the effects of these variables on temperature, melt pool, stress outputs, and potential defects, as well as geometry and layer number, are revealed, contributing to the improvement in scanning strategy and geometry design.

**Figure 1:**
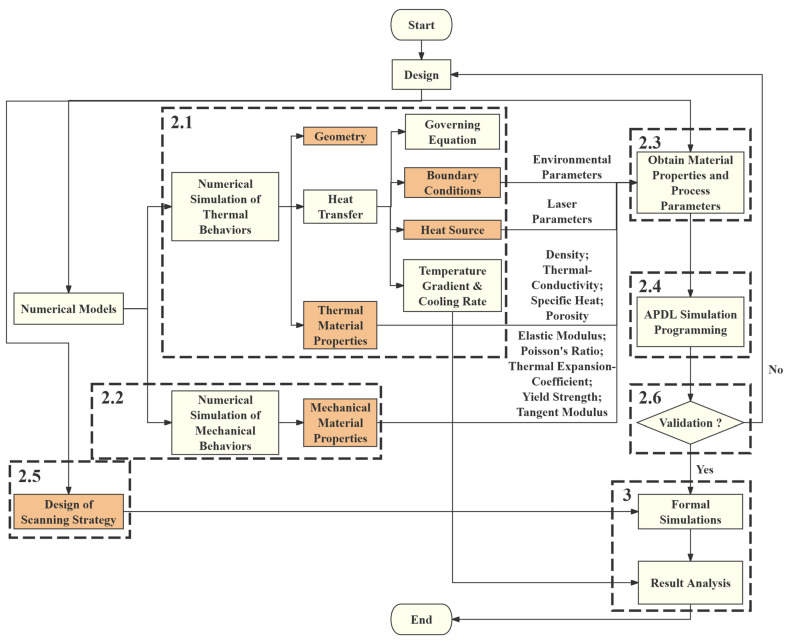
Procedures of the experiment; steps highlighted with orange have crucial impacts on the final results based on input data and steps corresponding to subsequent sections are also highlighted.

The rest of this paper is organized as follows: In the Materials and Methods section, the realization process of the FEA simulation is described, including the numerical models for thermal and mechanical behaviors, the obtaining of WE43 material properties and SLM process parameters, the procedures of the APDL simulation, and the design of scanning strategies. Additionally, a validation process for this simulation method is also conducted. In the Results and Discussion section, outputs regarding temperature, melt pool, and stress are gathered and compared, and patterns and characteristics are identified. Finally, in the Conclusion section, these patterns and characteristics are generalized and listed. Figure 1 shows the procedures of the experiment; steps highlighted in orange have crucial impacts on the final results based on the input data and steps corresponding to subsequent sections are also highlighted.

## 2. Materials and Methods

### 2.1. Numerical Simulation of Thermal Behaviors

#### 2.1.1. Assumptions

First of all, when conducting finite element analysis, the following assumptions would be made (all referenced assumptions are from [29]):

- The material is isotropic.
- In order to simplify the calculation, the shrinkage of the powder layer is ignored.
- The surface of the molten pool is assumed to be flat without respect to evaporation and capillary flow.
- The powder bed is treated as a homogeneous medium, and heat transfer between the powder pores is not considered.
- The convection coefficient between the powder bed and the forming chamber environment is constant.

#### 2.1.2. Geometry Model

**Figure 2:**
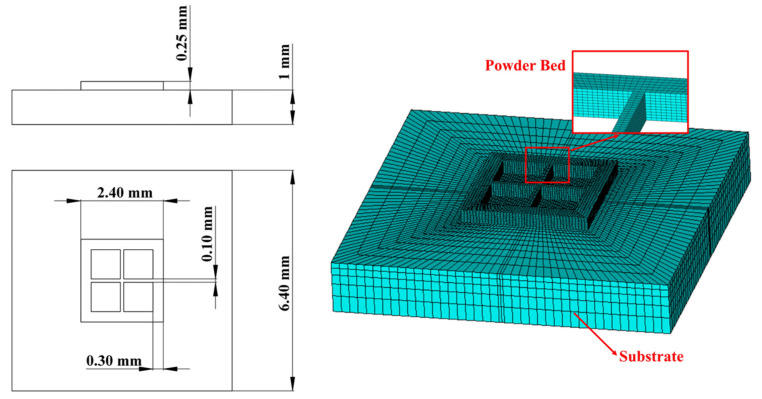
Simulated geometry.

The geometry model of an SLM process can be divided into two parts: the substrate part and powder bed part. The thermal analysis element that could be used here is SOLID70 or SOLID90 in Ansys APDL [29]. In this paper’s research, the reference geometry [11] was simplified to only show its main features and scaled down to quicken the simulation process, as Figure 2 shows.

The powder bed includes five powder layers, and each of them was sliced into 2 in terms of height; the length and width of the powder bed were divided into 42 sections each; the thickness of the outer frame was divided into 6 sections and that of the inner frame was divided into 2 sections. The substrate was divided by 10 in terms of height, with element size increasing from top to bottom. The thermal analysis element that was used was Solid70.

#### 2.1.3. Governing Equation

According to [51], based on previous assumptions, the governing equation of the heat transfer process can be written as:

(1) $$\rho c\frac{\partial T(x,y,z,t)}{\partial t}=\nabla \cdot [k\nabla T(x,y,z,t)]+Q(x,y,z,t)$$

or in another form, as follows [29]:

$$\rho c\frac{\partial T}{\partial t}=Q+\frac{\partial }{\partial x}\left(k\frac{\partial T}{\partial x}\right)+\frac{\partial }{\partial y}\left(k\frac{\partial T}{\partial y}\right)+\frac{\partial }{\partial z}\left(k\frac{\partial T}{\partial z}\right)$$

where *ρ* is the density, *c* is the specific heat capacity, *T* is the temperature in the model, *t* is the time of laser–powder interaction, *k* is the thermal conductivity, and *Q* is the heat generated per volume within the component.

#### 2.1.4. Boundary Conditions

First, the initial temperature $T_{0}$ (uniformly distributed throughout the powder bed, which is equal to the ambient temperature) can be written as [29]:

(2) $$T(x,y,z,t){|}_{t=0}=T_{0}$$

**Figure 3:**
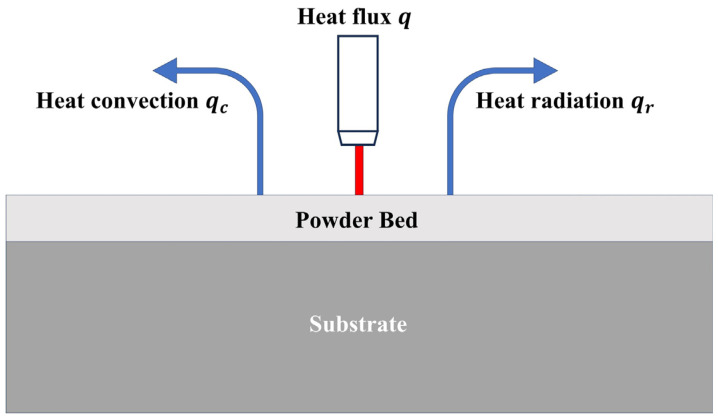
Boundary conditions.

On the top surface, the natural boundary condition can be expressed by (Figure 3) [29]:

(3) $$k\frac{\partial T}{\partial n}=q-q_{c}-q_{r}$$

where *n* is the normal vector of the surface, which is attached to the imposed heat fluxes; *q* is the heat supply; $q_{c}$ is the heat convection [29]:

(4) $$q_{c}=h_{c}(T-T_{0})$$

and $q_{r}$ is the heat radiation [29]:

(5) $$q_{r}=\sigma \varepsilon (T^{4}-T_{0}^{4})$$

where $h_{c}$ is the convective heat transfer coefficient, *σ* is the Stefan–Boltzmann constant, and *ε* is the surface radiation coefficient. Therefore, the previous equation can be written as [29]:

(6) $$k\frac{\partial T}{\partial n}=q-h_{c}(T-T_{0})-\sigma \varepsilon (T^{4}-T_{0}^{4})$$

#### 2.1.5. Heat Source

Based on [30], the heat flux from the laser head is usually simulated as a Gaussian heat source. For SLM, the common method is to assume a 2D Gaussian distribution:

(7) $$q=\frac{2\eta P}{\pi R^{2}}\exp\left(-2\frac{r^{2}}{R^{2}}\right)=\frac{2\eta P}{\pi R^{2}}\exp\left(-2\frac{x^{2}+y^{2}}{R^{2}}\right)$$

where *P* is the laser power; (*x*, *y*) is the position of a point on powder bed surface in the coordinate relative to the center of the laser spot; *r* is the radial distance from a point to the center of the laser spot; *R* is the effective laser beam radius at which the energy density is reduced to $1/e^{2}$ at the center of the laser spot; *η* is the effective absorption rate of powders to laser.

#### 2.1.6. Material Model

The effective thermal conductivity of the powder bed is influenced by the pores between the powders, the solid fraction, and the powder particle size. The powder bed porosity *φ* can be written as [29]:

(8) $$\varphi =\frac{\rho_{s}-\rho_{p}}{\rho_{s}}$$

where $\rho_{s}$ and $\rho_{p}$ are the density of the solid and powder bed, respectively.

The effective thermal conductivity of the powder bed can then be written as [29]:

(9) $$k_{p}=k_{s}(1-\varphi )$$

where $k_{s}$ and $k_{p}$ are the thermal conductivity of the solid and powder bed, respectively.

It is important to know that powders’ material properties would change when they turn into a solid, so in the simulation, when one of the node temperatures of a powder element exceeds its melting point, the properties of the element convert to the properties of a solid; if not, the properties of the powder remain.

The latent heat of phase transformation (melting and solidification during SLM) can be calculated using ANSYS by calculating the enthalpy *H* at different temperatures [29]:

(10) H=∫ρcdT

where *ρ* is the density and *c* is the special heat capacity.

#### 2.1.7. Temperature Gradient and Cooling Rate

Temperature gradient G(x,y,z) toward the *x*, *y,* and *z*-axes can be calculated as [29]:

$$G(x,y,z)=\frac{\partial T(x,y,z)}{\partial (x,y,z)}$$

The combined temperature gradient *G* is [29]:

(11) $$G=\sqrt{G_{x}^{2}+G_{y}^{2}+G_{z}^{2}}$$

The lager temperature gradient may lead to an increase in residual stress and cracking susceptibility during the SLM process.

The cooling rate $\dot{T}$ can be calculated from [29]:

(12) $$\dot{T}=-\frac{\partial T}{\partial t}$$

Generally, the greater the cooling rate of the molten pool, the finer the grain of the formed part and the better the comprehensive mechanical properties.

### 2.2. Numerical Simulation of Mechanical Behaviors

After the thermal analysis, a quasi-static mechanical analysis would be carried out using the current temperature field. First, the balance of linear momentum in any point of the body is given by the following [31]:

(13) div(σ)+b=0

where ***σ*** is the stress tensor and ***b*** is the body forces, which are neglected in this model. In terms of boundary conditions, displacements are imposed on the Dirichlet boundary; here, the substrate bottom is fixed during the whole SLM process.

To adopt a simplified ideal elastic–plastic hardening model, the total strain increment is given by the following [31]:

(14) $$\Delta \varepsilon^{total}=\Delta \varepsilon^{e}+\Delta \varepsilon^{p}+\Delta \varepsilon^{th}\;\Delta \varepsilon^{e}=\Delta \varepsilon^{total}-\Delta \varepsilon^{p}-\Delta \varepsilon^{th}$$

where $\Delta \varepsilon^{e}$ is the elastic strain increment, $\Delta \varepsilon^{p}$ is the plastic strain increment, and $\Delta \varepsilon^{th}$ is the thermal strain increment. The effects of strains induced by solid-state phase transformation and creep are neglected.

Based on the linear elastic constitutive law, the relationship between the stress tensor and the strain tensor is defined. Here, the resulting stress from the elastic strain is expressed as [31]:

(15) $$\sigma =C:\varepsilon^{e}$$

where ***C*** is the fourth-order material stiffness tensor (elastic moduli). Assuming an isotropic linear elastic material, the stiffness matrix ***C*** can be calculated from the Young’s modulus (*E*) and the Poisson’s ratio (*ν*).

Considering an associated flow rule in the plasticity model, the plastic strain increment is given by [31]:

(16) $$\Delta \varepsilon^{p}=\lambda \frac{\partial f}{\partial \sigma }$$

where *λ* is the plastic multiplier, which is calculated through the consistency condition. The plastic behavior is also assumed to be isotropic and described by the von Mises yield criteria [35]. Hence, the yield function *f* is obtained by [31]:

(17) $$\sigma_{vM}=\frac{\sqrt{2}}{2}\sqrt{{\left(\sigma_{1}-\sigma_{2}\right)}^{2}+{\left(\sigma_{2}-\sigma_{3}\right)}^{2}+{\left(\sigma_{3}-\sigma_{1}\right)}^{2}}$$

(18) $$f=\sigma_{vM}-\sigma_{y}$$

where $\sigma_{vM}$ represents the von Mises equivalent stress and $\sigma_{y}$ is the yield stress. $\sigma_{1}$, $\sigma_{2}$, and $\sigma_{3}$ are the three principal stresses in mutually perpendicular directions.

The phenomenological Swift law can be adopted to describe the hardening of the material. The isotropic work hardening function is given by the following [31]:

(19) $$\sigma_{y}=K{\left(\varepsilon_{0}+{\bar{\varepsilon }}^{p}\right)}^{n}$$

where *K*, $\varepsilon_{0}$, and *n* are the material parameters, while ${\bar{\varepsilon }}^{p}$ denotes the equivalent plastic strain. The initial yield stress is defined by $\sigma_{y}=K{\left(\varepsilon_{0}\right)}^{n}$.

The total thermal strain is calculated as [35]:

$$\varepsilon^{th}=\int_{T_{ini}}^{T}\alpha (T)dT$$

where *α* is the coefficient of the thermal expansion of the material, presented in a simpler form as follows [31]:

(20) $$\varepsilon^{th}=(\alpha_{T}(T-T_{ref})-\alpha_{ini}(T_{ini}-T_{ref}))I$$

where $\alpha_{T}$ and $\alpha_{ini}$ are the volumetric thermal expansion coefficients evaluated at the current temperature *T* and at the initial temperature $T_{ini}$, respectively. $T_{ref}$ is the reference temperature used to define the thermal expansion coefficients and ***I*** denotes the second-order identity tensor.

For practical applications, what we are concerned with is actually residual stress, which is the stress remaining in the product when it is cooled down to room temperature. Residual stress can have a detrimental influence on dimensional accuracy (particularly in thin-walled features) and mechanical performance, and is greatly influenced by the scanning strategy [42].

### 2.3. Material Properties and Process Parameters

As referenced in the study of Soderlind Julie et al. [48], the composition of WE43 is shown in Table 1.

It is notable that, in real experiments, there could be some O element in its composition due to oxidation (forming MgO and Y_2_O_3_).

**Figure 4:**
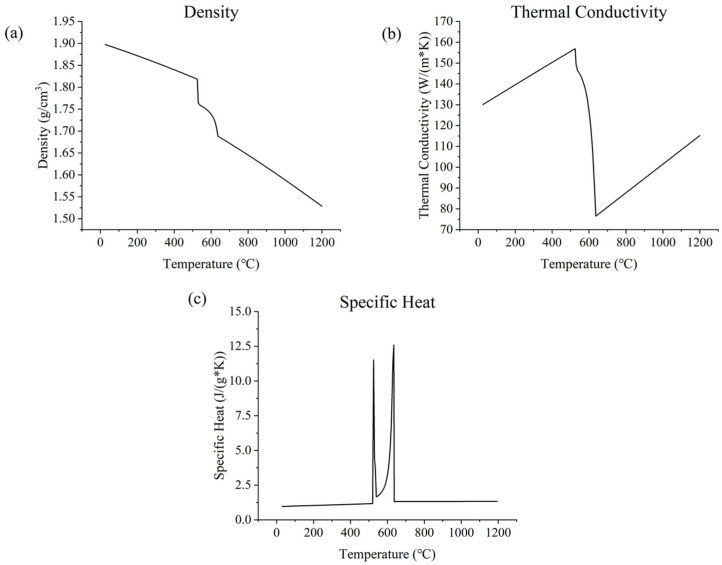
Thermophysical properties of WE43: (**a**) density; (**b**) thermal conductivity; (**c**) specific heat. Obtained from JMatPro 7.0 software by specifying the element composition.

The thermophysical properties (density, thermal conductivity, and specific heat) of WE43 may be obtained from JMatPro 7.0 software by specifying the element composition (1%wt RE element could be integrated into Nd, as [48] indicated). Thus, the properties of WE43 from 25 °C to 1200 °C could be observed (Figure 4). It can be seen that the values of the thermophysical properties of WE43 tend to undergo rapid changes around melting point, e.g., a rapid drop in density and thermal conductivity and a rapid rise and fall in specific heat, but the rest of the parts basically remain linear (in spite of the rapid changes due to melting; with the increase in temperature, density tends to decrease, thermal conductivity tends to increase, and specific heat remains constant). The results obtained from the software generally agree with the results of others’ research [49] and in the engineering handbooks [52,53], although some larger variances in thermal conductivity could be observed due to the difference in specific element compositions [54].

The melting temperature of WE43 could also be found using JMatPro, $T_{m}=525\;{}^{\circ}C$, where liquid fraction occurs. As mentioned previously, H=∫ρcdT, enthalpy per unit volume can be approximately calculated by T×ρ×c. It is notable that the value of thermal conductivity and density ($\rho_{p}=\rho_{s}(1-\varphi )$) could be affected by the porosity *φ* of powders; according to [31], for metallic powders, thermal conductivity is roughly 10 times smaller than that of the same bulk material. Based on the equation $k_{p}=k_{s}(1-\varphi )$, a 90% porosity could be calculated. Another important factor is the anisotropy enhancement factor [55] for thermal conductivity: when above the melting point temperature, an anisotropy enhancement factor should be set for the material’s thermal conductivity to reduce the effect of neglecting the complex flow in the melt pool and the recoil pressure on the temperature field; thus, more accurate melt pool results could be obtained [56], although this may not be applicable in all scenarios [57]. This factor is usually linearly proportional to $P/\sqrt{V}$, but detailed coefficients should be measured experimentally [30]. In general, this factor ranges from 2 to 20; this work employed a factor of 10.

In terms of the laser applied in the SLM process, this paper referred to the work of [48]. They applied two kinds of lasers, with a radius of 25 μm and 40 μm, respectively, and conducted single track experiments with various powers and scanning speeds. The absorptivity of WE43 varies with laser power and melt pool depth, mainly due to the differences in conduction and keyhole modes [48]. In this work, calibrated via comparison between numerical and experimental results, as also referenced in [58], for a melt pool depth smaller than 150 μm (mainly when R=40 μm), an absorptivity of 0.15 would be taken, while for a larger melt pool depth (mainly when R=25 μm), an absorptivity of 0.65 was taken. Other parameters were obtained from [39], such as a convective heat transfer coefficient $h_{c}=15{\;W/(m}^{2}K)$ (with Ar), and emissivity ε=0.4. The power layer thickness was set as 50 μm; the substrate was of the same material as the powders.

After the thermal analysis, a mechanical analysis was conducted to obtain the residual stress field, and this required the temperature-dependent mechanical properties of WE43: elastic modulus (Young’s modulus), Poisson’s ratio, and thermal expansion coefficient [31]. These can also be obtained via calculation in JMatPro. Moreover, to consider bilinear kinematic hardening, the model also needs to include yield strength and tangent modulus under different temperatures [59], which could be obtained from [50] using the 0.2% offset method.

### 2.4. Procedures of Simulation

**Figure 5:**
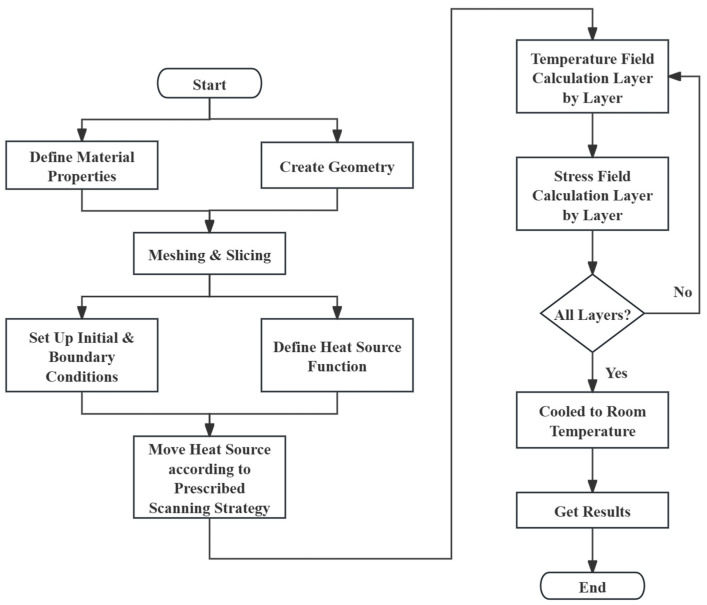
Procedures of simulation.

The basic procedures are shown in Figure 5 and were programmed in Ansys Mechanical APDL 2023 R1. Some important ones are as follows:

- **Material Properties:** The thermophysical properties of predefined materials were input, with a total of three materials being defined, all of which are WE43. Material 1 represents solid WE43, formed when metallic powders are melted by a laser and then solidify into a dense solid. Material 2 represents WE43 powders in their initial form as a powder bed, where porosity significantly influences their properties, causing them to vary from those of the other materials. Material 3 represents the substrate material, which is the same as solid WE43 but specifically used to represent the substrate.
- **Heat Source Function:** In this work, a 2D Gaussian heat source was used to simulate the laser. First, a heat source function was assigned to a local coordinate system. Then, the local coordinate system moved according to the prescribed scanning strategy during the simulation, applying and then deleting the heat source in each small step. Since it is a 2D Gaussian heat source, it was only applied to the top surface of each powder layer. Notably, this program used a technique called “Kill & Live” elements. When the heat source scans a certain powder layer, the powder layers above this layer are “killed” as if they do not exist, because, in reality, the next layer is only laid after the current layer’s scanning is finished. For the same reason, only after the scanning of the current layer is completed are the elements in the layer above “lived”.
- **Thermal–Mechanical Coupled Calculation:** Based on the elaborated models, the FEA software first calculates the temperature field. Only after the thermal calculation is completed can the mechanical calculation be performed based on the temperature field data. It is crucial to cool the model to room temperature in the final step, as this is when residual stress can be measured.

### 2.5. Design of Scanning Strategy

**Figure 6:**
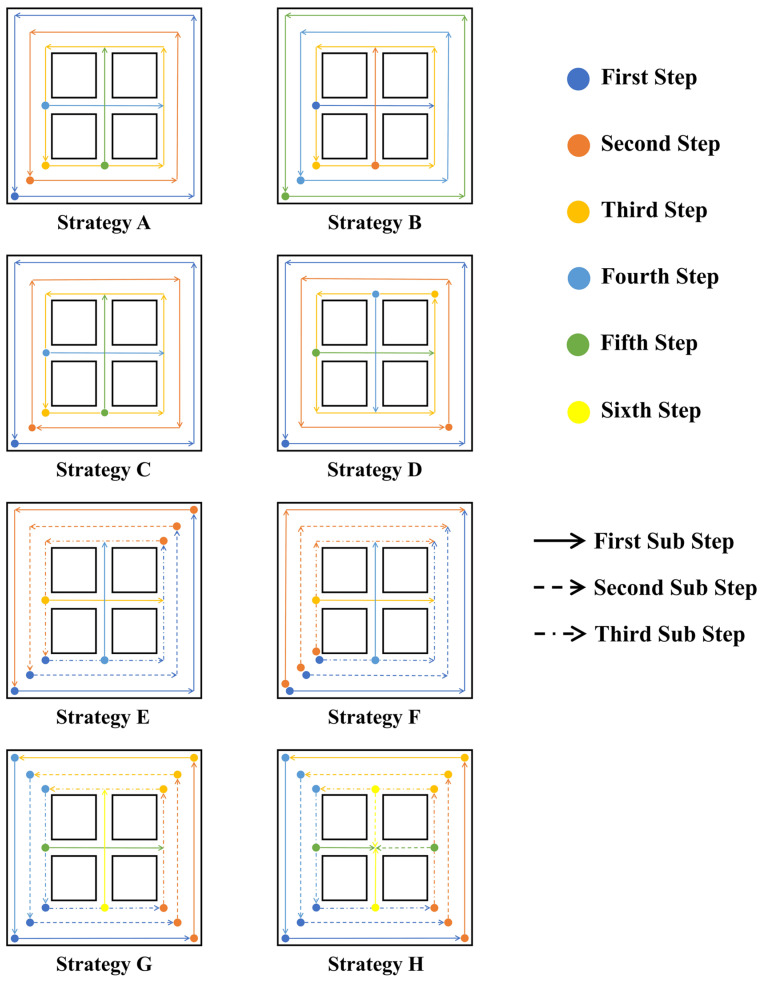
Strategies **A** to **H**.

Based on previous research, the possible influence of scanning strategy can be attributed to differences in scanning track length, direction, Out–In or In–Out strategy, start point, etc. [41]. To investigate the effects of these scanning strategy-related variables, a total of eight scanning strategies were designed, as shown in Figure 6. According to the current geometry, one layer can be divided into an outer frame and an inner frame. The outer frame is thicker and requires three scanning circulations, while the inner frame requires only one track. The designed scanning strategies avoid re-scanning the same point; for each circulation, the laser head ends a short distance from the start point instead of re-scanning it. Strategy **A** can be seen as the “default” scanning strategy, where the laser head scans the outer frame from the outer circulation to the inner circulation and scans the inner frame first horizontally and then vertically. In strategy **B**, the laser head scans the inner frame first and then scans the outer frame from the inner circulation to the outer circulation. By comparing the results with those of strategy **A** or **B**, the effects of the Out–In or In–Out strategy can be revealed. In strategy **C**, the laser head reverses its scanning direction for the next circulation of the outer frame, making it a bidirectional strategy. Comparing the results with those of strategy **A** or **C** can reveal the effect of changing scanning direction between circulations. In strategy **D**, the start point of the next circulation rotates to the next corner point, and the scanning of the inner frame is also rotated. Strategy **D** shows the effect of changing the start point between circulations. In strategy **E**, the laser head scans the bottom right part of the powder layer first and then scans the top left part from successive start points. Strategy **F** is similar to strategy **E**, but the scanning of the top left part starts from separated start points instead of successive ones. In strategy **G**, the laser head scans a quarter of the outer frame from the outside to inside each time. Strategy **H** is similar to strategy **G**, but the scanning of the inner frame is also divided into four steps. The results obtained with strategy **A**, **E**, or **G** together reveal the effect of changing the scanning circulation length (full, half, or quarter) on the outer frame. Comparing the results of strategies **E** and **F** shows the effect of continuous (same direction, successive start points) versus opposite (opposite direction, separated start points) scanning on the outer frame. Differences in the results of strategies **G** and **H** indicate the effect of sectional scanning on the inner frame.

**Figure 7:**
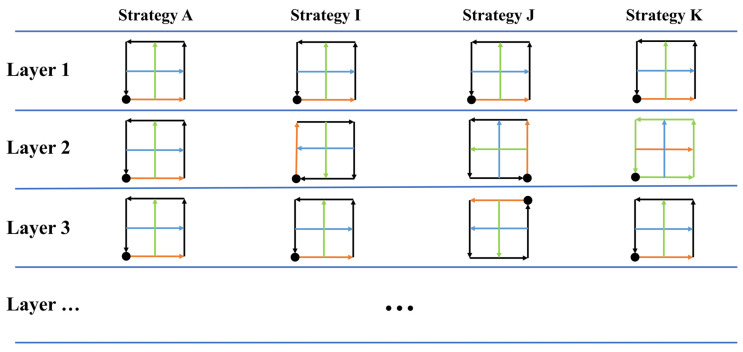
Strategies **A** and Strategies **I** to **K**.

Scanning strategies can vary not only within a single layer but also between different layers. Even if they have the same or a similar pattern, differences in the start point, direction, etc., can influence the final quality of the product [44]. Therefore, three more scanning strategies were designed, as shown in Figure 7. Strategy **I** reverses the scanning direction after every layer. Strategy **J** rotates the start point after every layer. Strategy **K** changes the Out–In or In–Out strategy after every layer. Compared with strategy **A**, strategies **I**, **J**, and **K** show the effects of changing the scanning direction, start point, and Out–In or In–Out strategy between layers, respectively.

**Figure 8:**
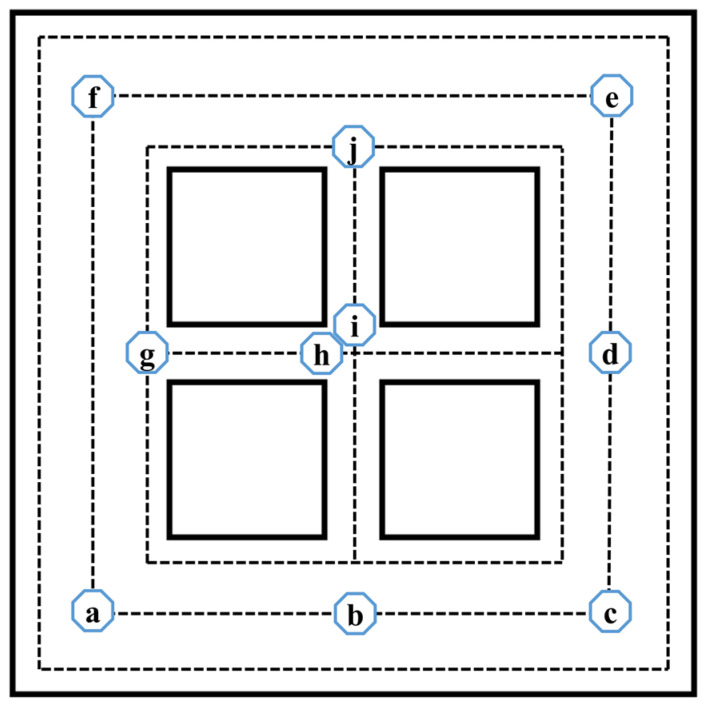
Testing points.

To reflect the effects of the scanning strategy-related variables, simulation outputs (e.g., melt pool dimensions, time-dependent temperature) were gathered from different testing points at certain times and using certain scanning strategies. These points are shown in Figure 8. Points **a**, **b**, **c**, **d**, **e**, and **f** are located in the center of the outer frame and primarily reflect the effect of the outer frame’s scanning strategy. Points **g**, **h**, **i**, and **j** are stress concentration points (where the cross-sectional area suddenly changes) in the inner frame and mainly reflect the effect of the inner frame’s scanning strategy. They can all reflect the effect of layer-dependent scanning strategies. However, it is notable that the effects of outer, inner, and layer-dependent scanning strategies can be coupled at certain locations.

### 2.6. Validation

**Figure 9:**
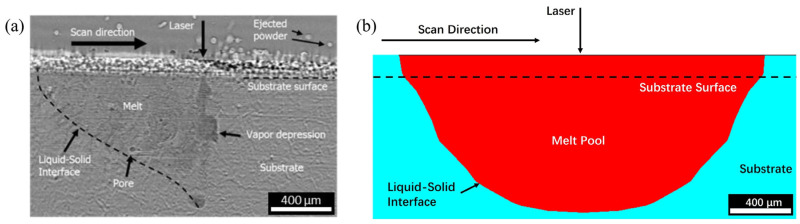
Melt pool formed when R=25 μm, P=150 W, and V=100 m m/s in (**a**) the single track experiment [48] and (**b**) the simulation program.

The reliability of the simulation program was validated by comparing its results with the experimental results from Soderlind Julie et al.’s work [48]. A comparison of the melt pools formed during the experiment and simulation is shown in Figure 9.

**Figure 10:**
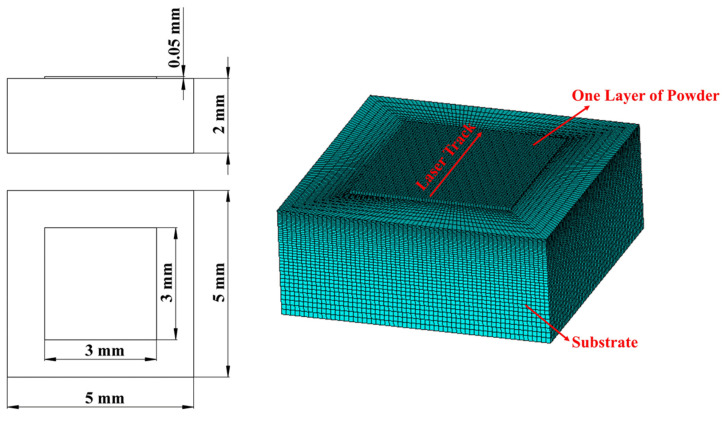
Validation geometry.

To conduct the simulation, a finite element geometry model for validation was created and meshed (Figure 10); the powder layer was 3×3×0.05 mm and meshed with 50×50×25 μm element size, while the substrate was 5×5×2 mm and divided into 40 sections in terms of height, with the element size increasing from top to the bottom. A laser heat source was applied and moved along the middle line of the powder layer with various powers and velocities to form melt pools with different depths.

**Figure 11:**
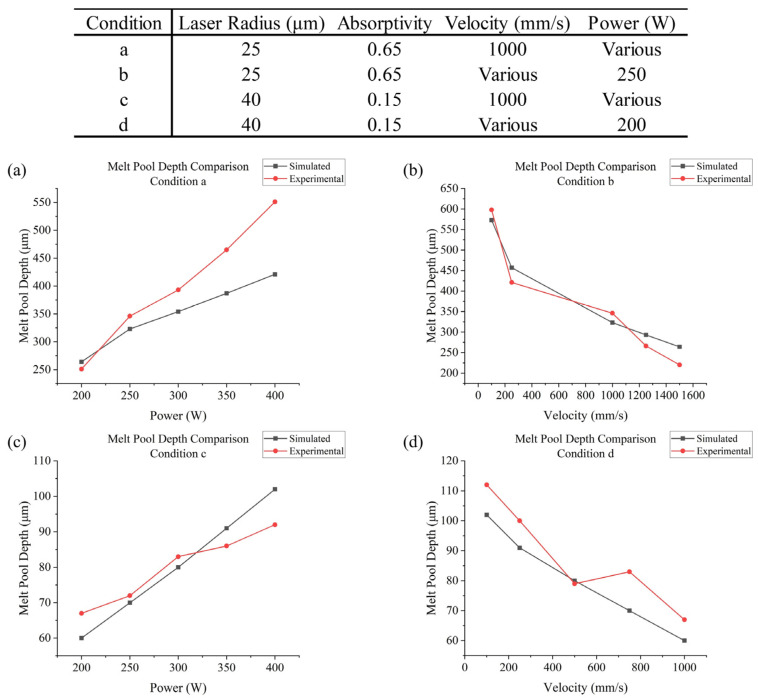
Melt pool depth comparison of the simulation and experiment (**a**) under condition **a**; (**b**) under condition **b**; (**c**) under condition **c**; (**d**) under condition **d**.

The validation process was conducted for two different kinds of lasers: one with a 25 μm radius and one with a 40 μm radius, as discussed previously, which have different absorptivities. The results are shown in Figure 11. They were measured using light-microscopy images of cross-sectioned laser tracks to evaluate the melt pool depths.

In conclusion, the simulation results basically match the experimental results within the current range of power and velocity (except the simulated melt pool depths, which tend to be lower for high-power 25 μm lasers), with an overall relative error of about 10%. Thus, the current material properties and process parameters are suitable for a scanning strategy analysis. The parameters used in this work’s simulation are generalized in Table 2.

## 3. Results and Discussion

### 3.1. Results

The simulation of SLM processes involved 11 different scanning strategies. In terms of temperature outputs, temperature and temperature gradient data can be directly exported from Ansys APDL. From the temperature data, time-dependent temperatures and peak temperatures at testing points in the fifth layer were obtained for strategies **A** to **H** (strategies differed within a layer). Additionally, the peak temperatures at these points across all layers were obtained for strategy **A** and strategies **I** to **K** (strategies differed between layers). Maximum cooling rates were calculated from a first-order derivative of temperature with respect to time. From the temperature gradient data, maximum temperature gradients were determined. Regarding melt pool outputs, the melt pool formed during the SLM process is defined as the volume at which the temperature exceeds the melting point (525 °C). The dimensions of the melt pool include its depth and width, which were measured at specific testing points. It is notable that each corner point (**a**, **c**, **e**, and **f**) connects two successive and perpendicular tracks, so their widths were measured in two directions. Melt pool life refers to the duration for which the temperature at a testing point remains above the melting point during laser scanning, and this can be determined from the temperature data. Regarding stress outputs, residual stress was measured after the model was cooled to room temperature, with significant changes observed during the cooling process. The X, Y, and Z components of stress, as well as the von Mises stress, their distributions, and maximum values, could be directly observed from the software. Additionally, the average von Mises stresses on the top surfaces of each layer for strategy **A** and strategies **I** to **K** could be calculated through simple programming within APDL.

### 3.2. Temperature Outputs Analysis

**Figure 12:**
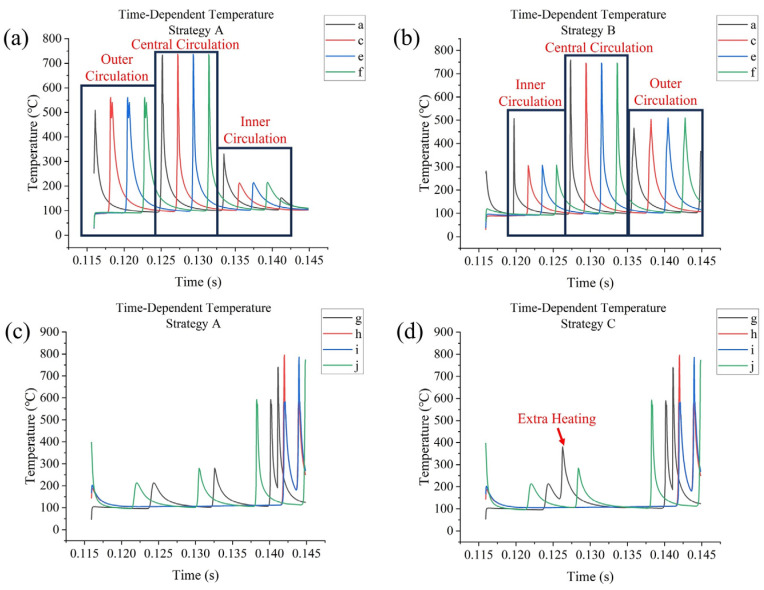
Time-dependent temperatures at (**a**) points **a**, **c**, **e**, and **f** for strategy **A**; (**b**) points **a**, **c**, **e**, and **f** for strategy **B**; (**c**) points **g**, **h**, **i**, and **j** for strategy **A**; (**d**) points **g**, **h**, **i**, and **j** for strategy **C**.

**Figure 13:**
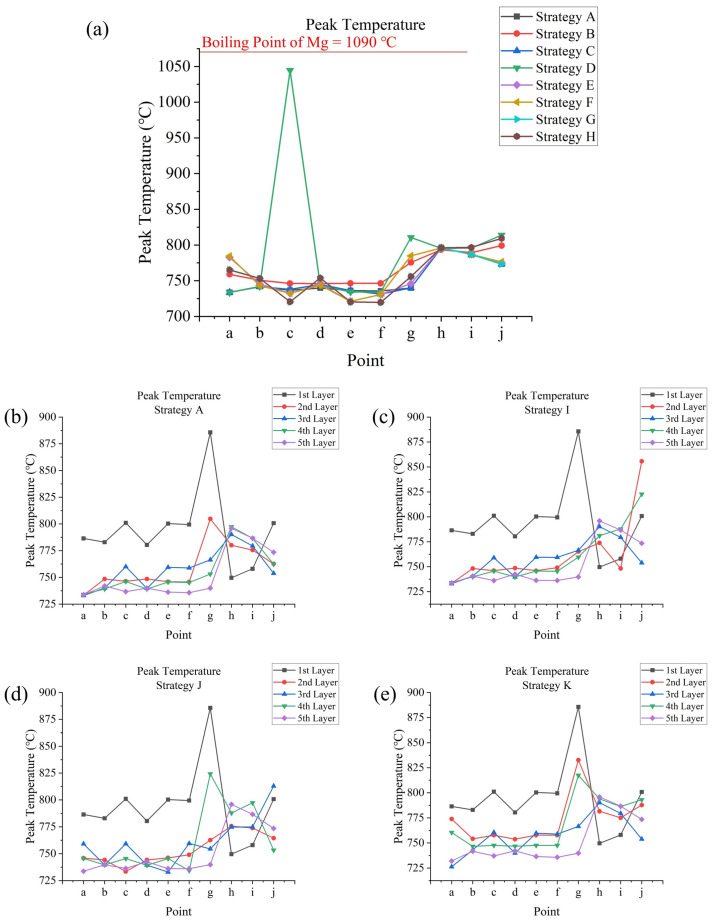
Peak temperatures at points **a** to **j** (**a**) in the fifth layer for strategies **A** to **H** and in all layers for (**b**) strategy **A**, (**c**) strategy **I**, (**d**) strategy **J**, (**e**) and strategy **K**.

The outer frame requires three circulations of scanning; each of them increases the temperature at testing points in the middle of the frame. For strategy **A**, as Figure 12a shows, the central circulation increased temperatures at the testing points the most significantly, to about 750 °C, since the laser head scanned them directly; temperature increases due to the outer circulation were the second highest, with temperatures increasing to about 550 °C; this could be due to the longer track length in the outer circulation, which allows for higher heat accumulation. Temperature increases due to the inner circulation were the most insignificant, reaching about 225 °C, but there was an extra temperature rise at point **a** due to the laser head returning close to point **a** at the end of the previous circulation. For the same reason, there was another slight temperature rise at the end of the inner circulation; however, this effect was not obvious on the outer and central circulations. Strategy **B** is an In–Out strategy, showing the effect of inner frame scanning (Figure 12b), which results in the additional heating of point **a** (to about 500 °C), as well as higher temperature rises at other testing points (to about 300 °C instead of 225 °C) due to the inner circulation; however, temperature rises due to the central and outer circulation were relatively uninfluenced. Strategy **C** indicates that the effect of changing direction between circulations is insignificant. Strategy **D** is a changing-start-point strategy, where the middle circulation starts at point **c** instead of point **a**, which thus causes an excessively high temperature at point **c**, increasing to about 1050 °C, which is very close to the boiling point of Mg, at 1090 °C, as Figure 13a shows, indicating that the sudden application of a heat source to a relatively cold part of the powder layer could lead to an excessively high temperature, increasing the risk of element loss due to evaporation.

The inner frame is a cross, containing points **g** and **j**, which connect the inner frame and outer frame. This would be influenced by the scanning of the outer frame. For most strategies, outer circulation would raise their temperatures to about 200 °C, central circulation raises their temperatures to about 250 °C, and inner circulation raises their temperatures to about 600 °C. Direct scanning from the laser head would increase the temperature at points **g** to **j** to about 800 °C, as Figure 12c shows. Temperature rise at point **g** due to central circulation was slightly higher for strategy **C**, reaching about 400 °C, since the laser head changed direction in the central circulation when it reached point **g** and the heat that accumulated due to outer circulation at this point was not yet fully dissipated (Figure 12d).

Peak temperatures occur at testing points when the laser head directly scans them. For points in the outer frame of the fifth layer, basically, their peak temperatures are about 750 °C, and for points in the inner frame, their peak temperatures are about 800 °C; this could be because the thinner width of the inner frame leads to an increase in heat accumulation. As for the effect of layer number, take strategy **A** as an example: as shown in Figure 13b, for different points, it might be different, and the first and second layer tend to provide eccentric results. This could due to the influence of the substrate. It is interesting that the data obtained from this research showed different phenomena compared to others’ research [29], where peak temperature generally increased with a higher layer number. The influencing factors could be geometry, material, powder layer thickness, etc.; for most points in the outer frame (**b** to **f**) and connection point **g**, the first layer tends to have very high peak temperatures, and from the third layer, the peak temperature decreases with an increase in layer number, while the second layer sometimes has a lower peak temperature. Point **a** is the start point in each layer; it has a higher peak temperature in the first layer and for the rest of the layers, its peak temperature basically shows no change. For points in the center of the inner frame (**h** and **i**), peak temperature increases with higher layer number, and for connection point **j**, its peak temperature increases with layer number from the third layer but the first and second layers provide eccentric results. In general, the change in peak temperature tends to decrease with increasing layer number; for example, at point **g**, peak temperature decreased by 81°C from the first to the second layer, decreased by 38°C from the second to the third layer, and decreased by about 13°C from the third to the fourth and from the fourth to the fifth layer.

For strategies with different scanning strategies in each layer (strategies **I**, **J**, and **K**), peak temperatures at testing points are not only influenced by layer number, but also by the scanning strategy used in this layer. Strategy **I** (Figure 13c) changes scanning direction every layer; its influence is greatest on points **g** and **j** and for layers where point **g** is the end of the inner scanning track and point **j** is the start point of the inner scanning track (second and fourth layers); point **g** tends to have lower peak temperatures, while point **j** tends to have higher peak temperatures. Strategy **J** (Figure 13d) changes start points every layer; in general, start points (point **a** for the first and fifth layers, point **c** for the second layer, point **e** for the third layer and point **f** for the fifth layer) of the outer frame scanning tend to have a lower peak temperature. Strategy **K** (Figure 13e) changes its Out–In or In–Out strategy every layer; for the In–Out strategy (second and fourth layers), points **g** and **j** have higher peak temperatures; point **a**, as the start point of the outer frame scanning, shows a higher variance in peak temperature compared with other strategies where **a** also works as concentrated start point.

**Figure 14:**
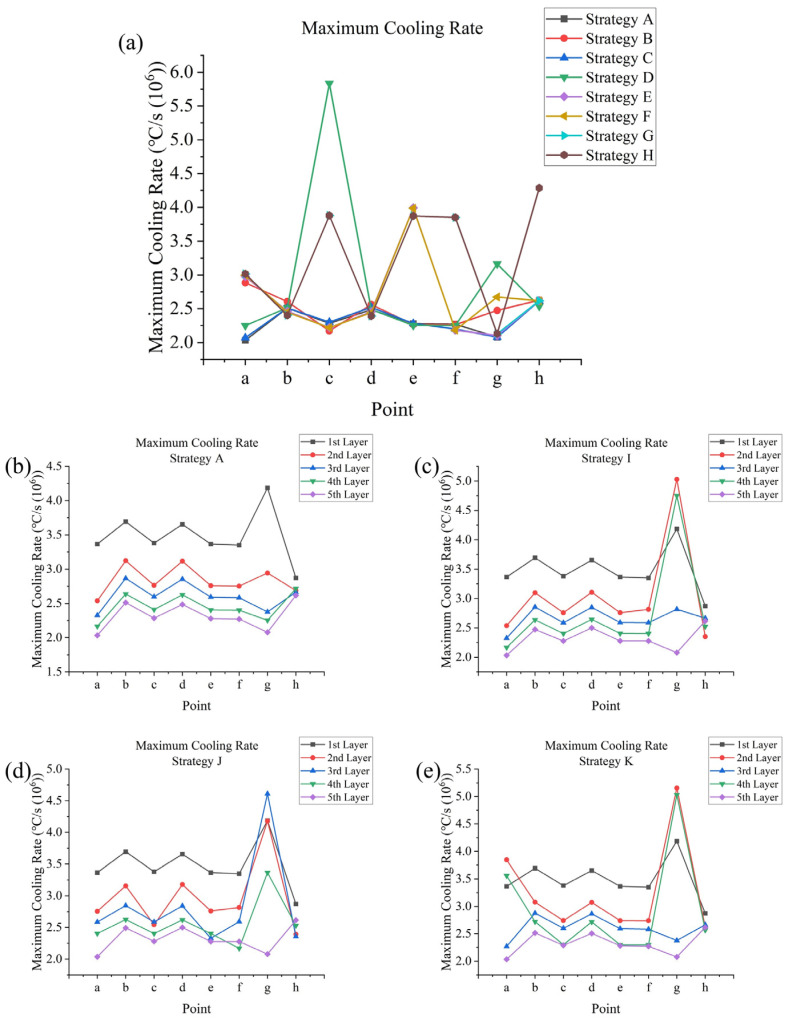
Maximum cooling rate at points **a** to **h** (**a**) in the fifth layer for strategies **A** to **H** and in all layers for (**b**) strategy **A**, (**c**) strategy **I**, (**d**) strategy **J**, (**e**) and strategy **K**.

The start point of a non-successive circulation or track (such as point **c** of strategy **D**, and point **a** of strategies **E** to **H**) and the end point of a non-successive track (such as point **e** of strategies **E** and **F**, points **c**, **e**, and **f** of strategies **G** and **H**, and point **h** of strategy **H**) tend to have higher maximum cooling rates; for strategy **B**, although point **a** is part of successive circulations, it still has a high maximum cooling rate of about $2.9\times 10^{6}\;{}^{\circ}C/s$ compared with strategy **A**, which has a cooling rate of about $2.0\times 10^{6}\;{}^{\circ}C/s$. This could be due to the heating of the outer frame from previous inner frame scannings being relatively less significant, thus leading to faster cooling. Connection point **g** has a high maximum cooling rate in strategy **B**, at $2.5\times 10^{6}\;{}^{\circ}C/s$, where it is the start point of the whole scanning process; thus, it does not undergo pre-heating from the outer frame scanning. In strategy **D**, the cooling rate is $3.2\times 10^{6}\;{}^{\circ}C/s$, and in strategy **F**, it is $2.7\times 10^{6}\;{}^{\circ}C/s$. In these strategies, the previous scanning ends far away from this point, thus there is less heating around this area. Center point of inner frame **h** and points at the centers of edges **b** and **d** tend to have a higher maximum cooling rate of about $2.4\times 10^{6}-2.6\times 10^{6}\;{}^{\circ}C/s$ compared with other points, where the cooling rate is about $2.0\times 10^{6}-2.3\times 10^{6}\;{}^{\circ}C/s$; this could be due to the thinner geometry at these points, which facilitates their cooling, as shown in Figure 14a.

As shown clearly in Figure 14b, the maximum cooling rate decreases with an increase in layer number, while the magnitude of change tends to decrease; cooling rates in the first layer are significantly high, which could be due to the heat dissipation caused by the substrate; Figure 14c,d indicate that point **g** would have a higher cooling rate as it is the end of a non-successive track rather than the start point of a non-successive track. Figure 14e indicates that when using the In–Out strategy (second and fourth layers), point **a** and point **g** would have higher cooling rates due to the lack of pre-heating from previous scannings. These effects could be very significant, thus compensating for the decrease due to the higher layer number and leading to a higher magnitude compared with the previous layer.

**Figure 15:**
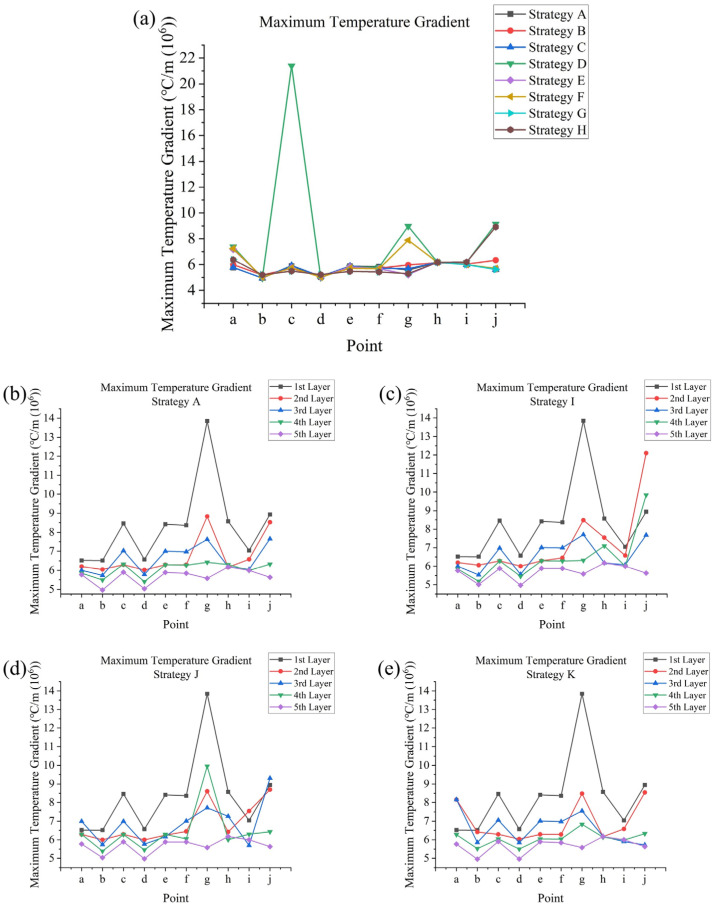
Maximum temperature gradient at points **a** to **j** (**a**) in the fifth layer for strategies **A** to **H**; and in all layers for (**b**) strategy **A**, (**c**) strategy **I**, (**d**) strategy **J**, and (**e**) strategy **K**.

The temperature gradient indicates the difference between the temperature at the testing point and the surrounding area, which depends on the overall average layer temperature increase and the effect of the previous scanning [41]. In general, a full circulation of scanning would lead to a higher average layer temperature, thus resulting in lower maximum temperature gradients at certain points; for example, point **b** in strategy **A**, where the laser head scans a full circulation at one time, has a maximum temperature gradient of about $5.0\times 10^{6}\;{}^{\circ}C/m$, but in strategy **G**, where the laser head scans with a quarter circulation at one time, this value is increased to $5.2\times 10^{6}\;{}^{\circ}C/m$. Furthermore, starting scanning at a point that is far away from the end of the previous scanning track would also lead to a higher temperature gradient; for example, at point **a**, strategy **E** leads to a maximum temperature gradient of about $7.2\times 10^{6}\;{}^{\circ}C/m$, while strategy **G** leads to a value of about $6.4\times 10^{6}\;{}^{\circ}C/m$, since a quarter-circulation scanning has a greater influence on its start point compared with a half-circulation scanning; however, for a full-circulation scanning, as in strategy **A**, since the laser head returns close to point **a** in the end, and there is a higher average layer temperature, point **a** has a low maximum temperature gradient, about $5.8\times 10^{6}\;{}^{\circ}C/m$. For the same reason, a corner point like point **c** tends to have a lower maximum temperature gradient with partial-circulation scanning: in strategy **A**, its value is $5.9\times 10^{6}\;{}^{\circ}C/m$, in strategy **E**, its value is $5.7\times 10^{6}\;{}^{\circ}C/m$, and in strategy **G**, its value is $5.5\times 10^{6}\;{}^{\circ}C/m$(as the end point of a non-successive track, it has a lower temperature gradient, although it also has a higher cooling rate). It is notable that the sudden application of a heat source to the colder part of the powder layer could lead to an excessively high maximum temperature gradient; point **c** of strategy **D** has a very high value of $21\times 10^{6}\;{}^{\circ}C/m$. These values are shown in Figure 15a.

In general, as Figure 15b shows, when using the same scanning strategy in each layer, the maximum temperature gradient has a tendency to decrease with an increase in layer number, and with the increase in layer number, this change tends to reduce. This could be due to the average layer temperature tending to increase with layer number due to the growing heat accumulation, but the second layer provided contradictory results, which were lower than expected. This could due to the effect of the substrate, which would cause the excessive heating of the first layer and thus significantly increase the average layer temperature of the second layer. Figure 15c–e show the combined effect of layer number and scanning strategy on each layer, which basically follows the rules mentioned before, i.e., for strategy **I**, the laser head starts scanning at point **j** in the second and fourth layers, and ends its scanning at point **j** in other layers, which leads to higher maximum temperature gradients at point **j** in the second and fourth layers.

### 3.3. Melt Pool Outputs Analysis

**Figure 16:**
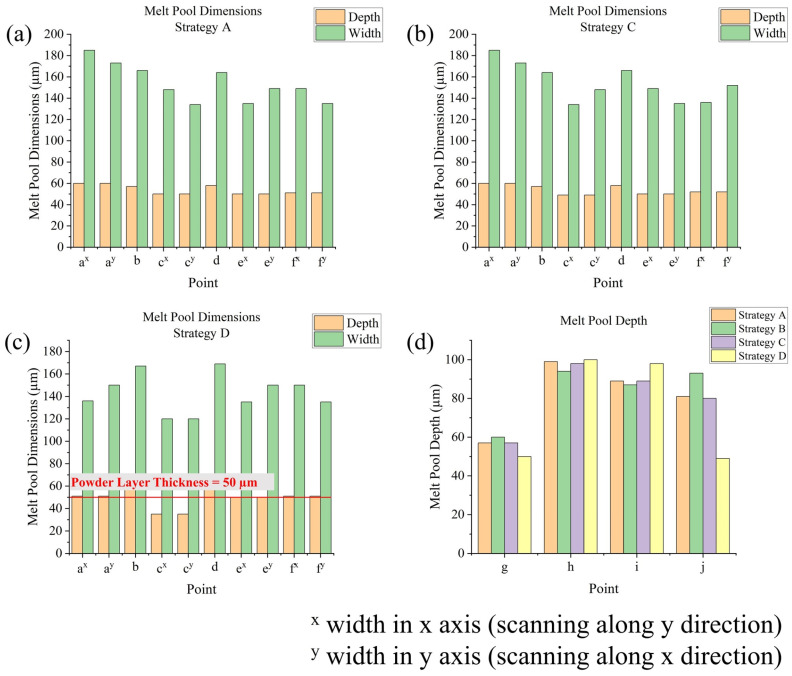
Melt pool dimensions at points **a** to **f** in the fifth layer for (**a**) strategy **A**, (**b**) strategy **C**, and (**c**) strategy **D**, and (**d**) melt pool depths at points **g** to **j** in the fifth layer for strategies **A** to **D**.

Melt pool dimensions include melt pool width and depth. Take strategy **A** as an example (Figure 16a): for points in the outer frame (**a** to **f**), depths range from 50 μm to 60 μm, while widths range from 130 μm to 190 μm, nearly three times the depths. This could be due to the low energy input, leading to very broad melt pools (limited conduction mode). For corner points (**a**, **c**, **e**, and **f**), start point **a** has higher melt pool dimensions (a 60 μm depth) compared with other corner points (about 50 μm depth), because point **a** is the start point of the successive circulation, meaning that the effect from the previous circulation is larger compared with that of the other corner points. Points in the middle of edges (points **b** and **d**) have higher melt pool dimensions compared with corner points (except point **a**), being about 7 μm deeper in depth and 20 μm wider in width, since the continuous direction of the scanning track (instead of changing directions at corners) would allow for more heat accumulation. For points in the inner frame (points **g** to **j**), points **i** to **j** have a much deeper depth (80 μm to 90 μm) than the middle and end points of the scanning track, which could be attributed to the thinner geometry of the inner frame, while point **g,** as the start point of the inner frame scanning track, where the effect of the previous circulation is relatively small, has a shorter melt pool depth (57 μm). At corner points, where two perpendicular tracks are connected, melt pool width is measured in two directions. For left and right edges, their directions move along the y axis; thus, their widths are measured along the x axis, while the opposite is true for the top and bottom edges. Generally, for a corner point, its width for the last track is smaller than its width for the next track. Take widths at point **c** in Figure 16a,b as a comparison: in strategy **A**, this point connects the top edge (width 134 μm) and the left edge (width 148 μm), while in strategy **C,** this point connects the left edge (width 134 μm) and the top edge (width 148 μm). If the start point of a new scanning is far away from the end point of the previous scanning, like the point **c** in Figure 16c, at this point there would be a very short melt pool depth of only 35 μm; it is notable that the powder layer thickness is 50 μm, indicating there is a risk of insufficient melting, and thus bad bonding. In strategy **D**, point **j** also has a short melt pool depth because, in this strategy, it is the start point of the inner scanning track instead of the end point. Strategy **B**, the In–Out strategy, would lead to higher melt pool widths at points **c**, **e**, and **f**, with an increase of about 20 μm, but lower melt pool widths at points **a**, **b,** and **d**, with a decrease of about 20 μm, showing the effect of inner frame scanning. However, for melt pool depths, its influence is relatively small: here, point **a** has a lower depth of about 50 μm, and the depths at point **g** and **j** slightly increased. Sectional scanning strategies **E** to **H** have a similar problem as strategy **D**; in these strategies, point **a** has low melt pool dimensions (depth of about 35 μm), due to the lack of the return of the laser head from the previous circulation, but the melt pool dimensions for the rest of the points are similar to those in strategy **A**.

**Figure 17:**
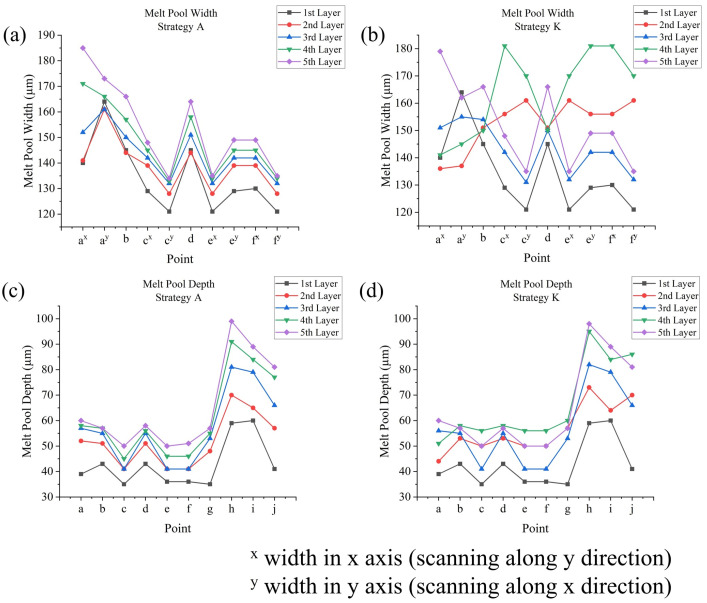
Melt pool widths at points **a** to **f** in all layers for (**a**) strategy **A** and (**b**) strategy **K**, and melt pool depths at points **a** to **j** in all layers for (**c**) strategy **A** and (**d**) strategy **K**.

As Figure 17a,c show, with the increase in layer number, melt pool dimensions (width and depth) also tend to increase. The magnitude of change varies with different testing points: in strategy **A**, from the first layer to the second layer, the y width of point **e** increased by 10 μm, but the width of point **d** decreased by 1 μm, although it increased in subsequent layers; in strategy **A**, for many points in the outer frame, from the first to the fifth layer, the widths increased by about 20 μm (most significant for x width of point **a**, which increased by about 50 μm) and the depths increased by about 20 μm; for points in the inner frame, the depths could be increased by about 40 μm. Changing the scanning strategy for each layer could also influence melt pool dimensions. Figure 17b,d shows the melt pool dimensions in all layers for strategy **K** (changes from Out–In or In–Out strategy each layer): the melt pool dimension variance basically follows the previously written rules; moreover, as can also be observed in the results of strategies **I** and **J**, the effect of the scanning strategy is more obviously shown in melt pool widths compared with melt pool depths, whose distribution and magnitudes are generally similar for different strategies. A melt pool is typically surrounded by a heat-affected zone (HAZ), where the temperature is not high enough to cause melting but can alter the mechanical properties and microstructure. The dimensions of the HAZ generally correspond to those of the melt pool (e.g., they also increase with higher layer numbers). Additionally, along the scanning direction, the HAZ behind the melt pool is larger than that in front of it [61].

**Figure 18:**
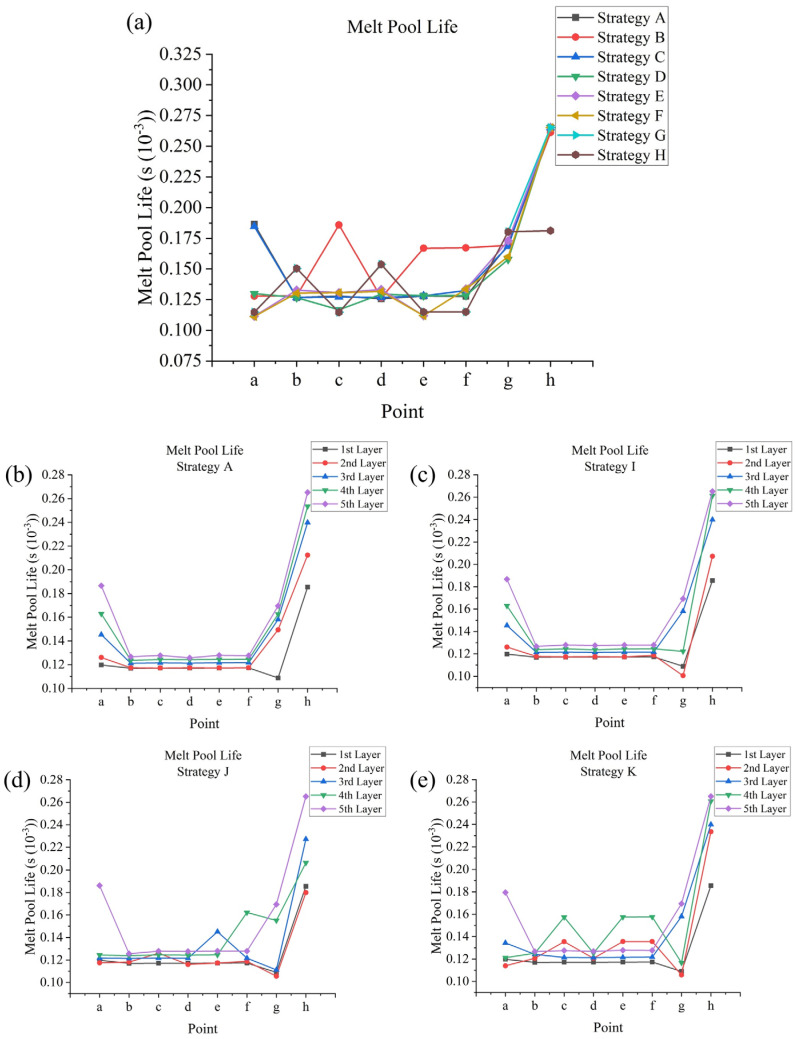
Melt pool lives at points **a** to **h** (**a**) in the fifth layer for strategies **A** to **H** and in all layers for (**b**) strategy **A**, (**c**) strategy **I**, (**d**) strategy **J**, and (**e**) strategy **K**.

Melt pool life is another important factor that could influence the final quality: too short a melt pool life would lead to poor wettability and a rough surface [62]. Melt pool life depends on the influence from the previous scanning. At those points where the effect of the previous scanning is large, melt pool life tends to be longer; for example, as Figure 18a shows, the start point of successive circulations, point **a** in strategy **A**, has a longer melt pool life of $0.18\times 10^{-3}\;s$, while most other points only have melt pool lives of about $0.13\times 10^{-3}\;s$. This is due to the laser head returning from its previous circulation; similar to melt pool dimensions, for an In–Out strategy (strategy **B**), the melt pool lives are larger at points **c**, **e**, and **f**, being increased by about $0.03\times 10^{-3}\;s$ to $0.06\times 10^{-3}\;s$. Additionally, point **b** in strategy **E** (over $0.13\times 10^{-3}\;s$) and strategy **G** (over $0.15\times 10^{-3}\;s$) has longer melt pool lives compared with those in strategy **A** due to the sectional scanning in strategy **E** and **G, which** causes concentrated heat accumulation in the scanned section. At those points where the effect of the previous scanning is small, like point **c** in strategy **D,** shorter melt pool lives are observed. Moreover, the start and end points of a non-successive track tend to have a shorter melt pool life, like points **a** and **e** in strategy **E** and points **a**, **c**, **e**, and **f** in strategy **G**, which have melt pool lives of about $0.11\times 10^{-3}\;s$ to $0.12\times 10^{-3}\;s$. Points in the inner frame have higher melt pool lives: the start point of the inner frame scanning track, point **g,** has a melt pool life of about $0.16\times 10^{-3}\;s$ to $0.18\times 10^{-3}\;s$; the middle point of an inner frame scanning track, point **h,** has a melt pool life of about $0.27\times 10^{-3}\;s$. However, when point h functions as the end point of scanning track, its melt pool life is reduced to $0.18\times 10^{-3}\;s$. Figure 18b–e shows the combined effect of layer number and scanning strategy: with the increase in layer number, melt pool life also tends to increase, but the magnitude of change varies for different points. For example, for start point **a** in strategy **A**, the melt pool life increased by about $0.01\times 10^{-3}\;s$ each time, but at other points, like point **b**, the increase was only $0.003\times 10^{-3}\;s$; the variance in melt pool lives for strategies **I** to **K** basically follows the rules written before.

### 3.4. Stress Outputs Analysis

**Figure 19:**
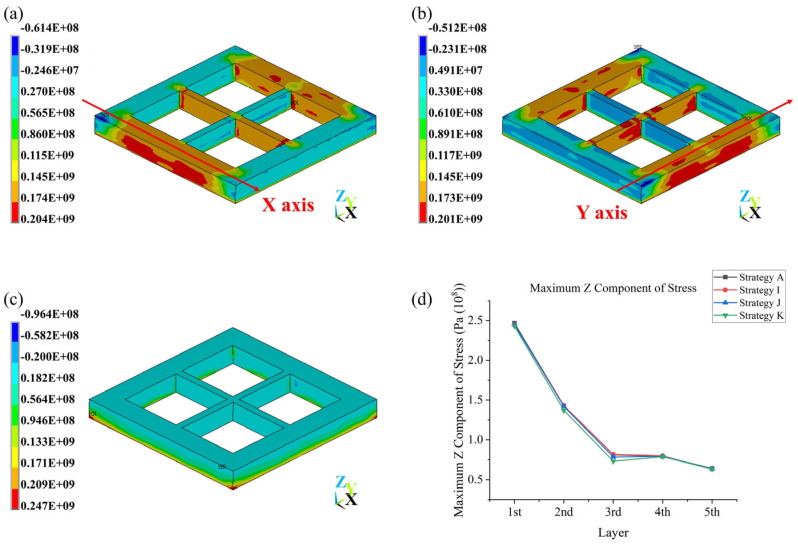
(**a**) X component of stress for strategy **A**; (**b**) Y component of stress of strategy **A**; (**c**) Z component of stress of strategy **A**; (**d**) maximum Z components of stress in different layers for strategy **A** and strategies **I** to **K**.

The distribution of the X, Y, and Z components of stress is similar for all strategies. Take strategy **A** as an example (Figure 19); in terms of the X component of stress, large-magnitude tensile stress (mainly ranges from $1.45\times 10^{8}\;\mathrm{Pa}$ to $1.74\times 10^{8}\;\mathrm{Pa}$) occurs in frames that were scanned along the x axis, and small-magnitude tensile and compressive stress (mainly range from $-2.46\times 10^{6}\;\mathrm{Pa}$ to $2.70\times 10^{7}\;\mathrm{Pa}$) occurs in frames that were scanned along the y axis. The Y component of stress shows the opposite phenomenon, with similar magnitude of stress. The Z component of stress presents a different situation: in the upper part of the geometry, the Z component of stress is mainly small-magnitude tensile and compressive stress, ranging from $-2.00\times 10^{7}\;\mathrm{Pa}$ to $1.82\times 10^{7}\;\mathrm{Pa}$, but in the lower part of the geometry, which is close to the substrate, the Z component of stress is mainly large-magnitude tensile stress, reaching up to $2.47\times 10^{8}\;\mathrm{Pa}$. The maximum Z component of stress tends to decrease with an increase in layer number; a significant decrease (from about $2.50\times 10^{8}\;\mathrm{Pa}$ to about $0.75\times 10^{8}\;\mathrm{Pa}$) was observed from the first layer to the third layer for strategy **A** and strategies **I** to **K**, but from the third layer above, there is basically no change in the maximum Z component of stress.

**Figure 20:**
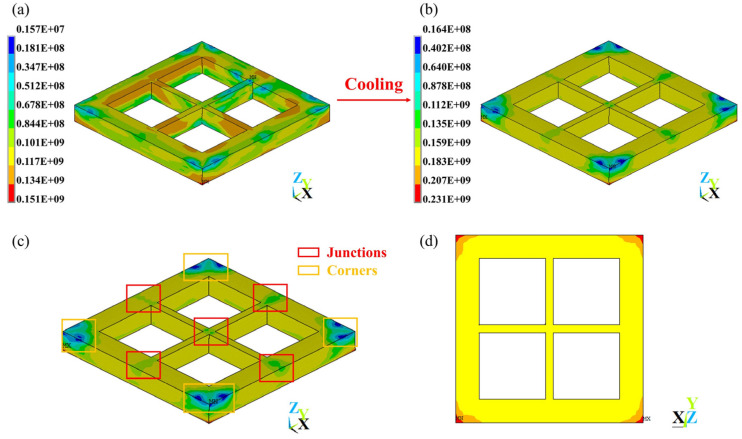
For strategy **A**: (**a**) von Mises stress just after scanning; (**b**) von Mises stress after cooling; (**c**) junctions and corners of geometry; (**d**) bottom surface of geometry.

As Figure 20a,b shows, just after scanning, the von Mises stress distribution was quite irregular, while during cooling, its distribution became more regular and the stress magnitude also increased (for strategy **A**, just after scanning, the geometry had a von Mises stress ranging from $1.57\times 10^{6}\;\mathrm{Pa}$ to $1.51\times 10^{8}\;\mathrm{Pa}$, while after cooling, the geometry had a von Mises stress ranging from $1.64\times 10^{7}\;\mathrm{Pa}$ to $2.31\times 10^{8}\;\mathrm{Pa}$). In terms of the final residual stress of strategy **A**, as Figure 20c,d shows, lower stress occurs in the junctions and corners of upper layers ($1.64\times 10^{7}\;\mathrm{Pa}$ to $1.59\times 10^{8}\;\mathrm{Pa}$), the main bulk of the geometry has a stress of $1.59\times 10^{8}\;\mathrm{Pa}$ to $1.83\times 10^{8}\;\mathrm{Pa}$, and larger stress occurs in the corners of the bottom layer ($1.83\times 10^{8}\;\mathrm{Pa}$ to $2.31\times 10^{8}\;\mathrm{Pa}$). This shows that the tensile stress at corners causes the bottom layer to have a warping tendency, but the constraint of the substrate causes stress to increase.

**Figure 21:**
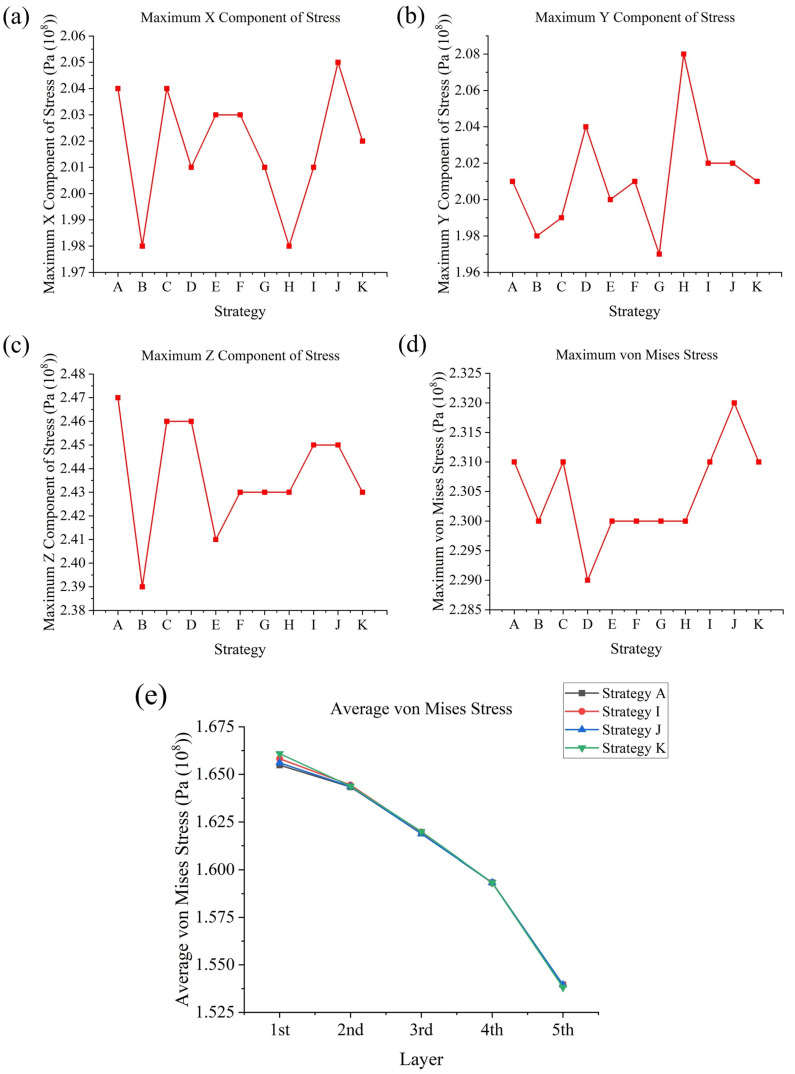
(**a**) Maximum X components of stress; (**b**) maximum Y components of stress; (**c**) maximum Z components of stress; (**d**) maximum von Mises stresses for different scanning strategies; (**e**) average von Mises stress in each layer for strategy **A** and strategies **I** to **K**.

All strategies have a similar stress distribution; thus, the conclusion could be drawn that the stress distribution is mainly determined by geometry rather than scanning strategy. However, with different scanning strategies, the magnitudes of stress could be changed, and a small amount of residual stress is desirable. In this work, as Figure 21a–d shows, among a total of 11 scanning strategies, the maximum X component of stress ranges from $1.98\times 10^{8}\;\mathrm{Pa}$ to $2.05\times 10^{8}\;\mathrm{Pa}$; the maximum Y component of stress ranges from $1.97\times 10^{8}\;\mathrm{Pa}$ to $2.08\times 10^{8}\;\mathrm{Pa}$; the maximum Z component of stress ranges from $2.39\times 10^{8}\;\mathrm{Pa}$ to $2.47\times 10^{8}\;\mathrm{Pa}$; and the maximum von Mises stress ranges from $2.29\times 10^{8}\;\mathrm{Pa}$ to $2.32\times 10^{8}\;\mathrm{Pa}$. Although larger variances were observed for the X, Y, and Z components of stress, there was less variance in the von Mises stress, which indicates that large variances in any of the X, Y, or Z components of stress do not necessarily mean that von Mises stress is very different. In conclusion, strategy **B** (an In–Out strategy) led to very low maximum X, Y, and Z components of stress and relatively low maximum von Mises stress; strategy **C** (a bidirectional strategy) did not vary a lot compared to strategy **A**; strategy **D** (a rotating start points strategy) did not show a large difference in maximum X, Y, Z components of stress but obtained the lowest maximum von Mises stress, which could due, apart from the high temperature gradients at some points, to this method resulting in a lower temperature non-uniformity overall, which is roughly proportional to the residual stress [41]. Half-sectional scanning strategies (strategies **E** and **F**) obtained relatively low maximum Z components of stress and von Mises stresses. Quarter-sectional scanning strategies (strategies **G** and **H**) also obtained relatively low maximum Z components of stress and von Mises stresses, but there was difference in the maximum X and Y components of stress between them, where strategy **G** has a higher maximum X component of stress and lower maximum Y component of stress, and strategy **H** has the opposite, showing the effect of the inner frame scanning strategy. Sectional scanning strategies reflected the combined effect of an increase in average layer temperature and cumulative tracks’ influence on temperature gradient and temperature non-uniformity. Strategies **I**, **J**, and **K** showed the effect of scanning strategies differing between layers, strategies changing direction or Out–In or In–Out strategy each layer could result in lower maximum X and Z components of stress; however, changing start points between layers could have a negative effect that leads to a higher maximum X component of stress and maximum von Mises stress. Furthermore, with the increase in height, the average von Mises stress for each layer tends to decrease, and the reduction each time tends to increase (Figure 21e).

## 4. Conclusions

Damping alloys are useful for noise and vibration suppression in engineering applications. A damper structure on electronic packages made with WE43 could be manufactured via selective laser melting (SLM). To gain a better understanding of the residual stress and potential defects induced by the manufacturing process, the effects of scanning strategy on the thermal and mechanical behaviors of SLMed products were investigated. In this work, SLM simulations, using a total of 11 different scanning strategies, were conducted, and their results, in terms of temperature, melt pool, and stress, were compared. The conclusions drawn from this study are as follows:

- **From temperature outputs analysis:** For a circulation scanning, the outer (longer) circulation has a more significant effect on the middle area compared to the inner (shorter) circulation, the sudden application of a heat source to a relatively colder part of the powder layer can lead to an excessively high peak temperature. The start point of a non-successive circulation or track and the end point of a non-successive track tend to exhibit higher maximum cooling rates, while the temperature gradient depends on the overall average layer temperature rise and the effect of previous scanning passes. Additionally, a thinner geometry leads to higher peak temperatures and cooling rates. The effect of a higher layer number on peak temperature varies at different points but generally results in lower cooling rates and temperature gradients, but the first and second layers may produce eccentric results. The results indicate that the inappropriate arrangement of scanning track length or start point, or the use of an Out–In or In–Out strategy, can lead to defects such as an undesired HAZ or element loss due to evaporation. Conversely, a well-designed scanning strategy can create a lower overall temperature gradient and a suitable cooling rate, both of which are desirable for better mechanical performance. The potential risks associated with thin-walled geometry and a higher layer number are also revealed, providing valuable insights for improving geometry design.
- **From melt pool outputs’ analysis: A** low energy input leads to a large width and low depth. Successive circulations, or tracks with a continuous direction, lead to larger melt pool dimensions at the connection points. For a corner point that connects two perpendicular tracks, the melt pool width for the last track is smaller than its width for the next track, and an In–Out strategy would lead to larger widths at certain points but have an insignificant influence on depth. The effect of the scanning strategy is more obvious on melt pool width compared with melt pool depth. Melt pool life depends on the influence from previous scannings; the start and end points of a non-successive track tend to have shorter melt pool lives. A thinner geometry or higher layer number lead to larger melt pool dimensions and a longer melt pool life. The noncontinuity of scanning direction or start point could lead to small melt pool dimensions and a short melt pool life, leading to the risk of poor bonding due to insufficient melting or a rough surface. It is notable that melt pool width is more susceptible to the difference in scanning strategy, and the low melt pool dimensions at lower layers should be specifically addressed.
- **From stress outputs’ analysis:** In terms of the X component of stress, large-magnitude tensile stress occurs in frames that were scanned along the x axis, while low-magnitude tensile and compressive stress occurs in frames that were scanned along the y axis; the opposite is true for the Y component of stress; the Z component of stress decreases from the bottom to the top surface. During cooling, von Mises stress becomes more regularly distributed and larger, smaller stress occurs in the junctions and corners of upper layers, and larger stress occurs in the corners of the bottom layer. Among all scanning strategies, although larger variances were observed in the X, Y, and Z components of stress, less variance was found in von Mises stress. In terms of von Mises stress, strategy **D** (changing the start point each circulation) led to the lowest value, followed by strategy **B** (In–Out strategy) and sectional scanning (strategies **E** to **H**). Changing start point each layer (strategy **J**) could lead to a higher magnitude of stress, and average von Mises stress decreases with height. The variances in the distributions of different kinds of stress suggest a potential method for enhancing overall mechanical performance by applying anisotropic materials. More attention should be paid to addressing defects occurring in the lower layers, as there is a tendency for the Z component of stress and the average von Mises stress to decrease with an increase in layer numbers. Changing the start point between circulations has both advantages and disadvantages: on the one hand, it leads to the lowest maximum von Mises stress, but on the other hand, it leads to excessively high peak temperatures and low melt pool depths at certain locations. In this way, a trade-off between favorable thermal characteristics and mechanical properties was demonstrated.
- **Commercial significance:** Although, compared with traditional manufacturing methods, SLM has advantages like near-net-shape production, no tool wearing, etc., this technique is still subjected to limitations like higher operating costs, small build volumes, and a low production rate; thus, it is not widely applied yet. S. Palanivel et al. [63] are exploring new ways to reduce the manufacturing costs. Moreover, the existence of RE content in WE43 also increases these costs; new, low-cost, high-strength WE43 alloys are under development [64]. Having said that, the application of SLMed WE43 is still promising and its production cost is decreasing; furthermore, with an improvement in the damping and the mechanical properties of manufactured parts, as well as an increase in the convenience of additive manufacturing for part repairs, the exploitation and maintenance costs could be significantly reduced.
- **Future research:** This paper demonstrates possibilities for future research: (a) In terms of SLMed WE43, more experiments could be conducted for further model verification and validation, such as an analysis of HAZ characteristics, a microstructure analysis using optical microscopy (OM) or scanning electron microscopy (SEM), an element distribution analysis using energy-dispersive X-ray spectroscopy (EDX), or a grain and phase analysis using electron backscatter diffraction (EBSD). [65] (b) Research on SLMed Mg-RE alloys is still seldom seen. Based on the current experiment method and conclusions, the characteristics of SLMed Mg-Gd, Mg-Nd alloys, etc., or other newly developed Non-RE Mg alloys with a superior damping capacity (e.g., Mg-Li alloys), can also be explored [10]. (c) Apart from a particle damper structure, more useful structures for aerospace [66], bio-application [67], robotics [68], etc., could be designed and manufactured with SLMed WE43, as well as with other Mg-RE alloys. (d) In addition to scanning strategy, the effects of other SLM parameters, like power, scanning velocity, porosity, pre-heating temperature, etc., on the thermal and mechanical behaviors of WE43 have yet to be investigated.

## Figures and Tables

**Table 1:** Elements in WE43 [48].

Element	Y	Nd	RE	Zr	Mg
Weight %	4	3	1	0.4	Margin

**Table 2:** Parameters of SLM.

Parameter	Value	Source
Laser Absorptivity, *η*	0.15	[60]
Laser Radius, *R* [μm]	40	[48]
Scanning Velocity, *V* [mm/s]	1000	-
Power, *P* [W]	200	-
Powder Layer Thickness [μm]	50	-
Convective Heat Transfer Coefficient, $h_{c}$ [${W/(m}^{2}K)$]	15	[39]
Radiation Emissivity, *ε*	0.4	[39]
The Stefan–Boltzmann Constant *σ* [${W/(m}^{2}K^{4})$]	$5.67\times 10^{-8}$	[39]

## Data Availability

Data are contained within the article.

## Nomenclature

SLM	Selective laser melting	*R*	Laser beam radius (μm)
AM	Additive manufacturing	*η*	Absorptivity
RE	Rare earth	*φ*	Porosity
FEA	Finite element analysis	*H*	Enthalpy (${J/m}^{3}$)
APDL	Ansys parametric design language	*G*	Thermal gradient (°C/m)
HAZ	Heat-affected zone	$\dot{T}$	Cooling rate (°C/s)
*ρ*	Density (${\mathrm{kg}/m}^{3}$)	***σ***	Stress tensor (Pa)
*c*	Specific heat (J/(kg∗K))	$\sigma_{vM}$	von Mises stress (Pa)
*k*	Thermal conductivity (W/(m∗K))	$\sigma_{y}$	Yield stress (Pa)
*T*	Temperature (°C)	$\sigma_{1,2,3}$	Principle stresses (Pa)
$T_{0}$	Initial temperature (°C)	***b***	Body forces (N)
$T_{m}$	Melting point (°C)	$\varepsilon^{total}$	Total strain
*Q*	Heat generated per volume (${W/m}^{3}$)	$\varepsilon^{e}$	Elastic strain
*q*	Heat flux (W)	$\varepsilon^{p}$	Plastic strain
$q_{c}$	Heat convection (W)	$\varepsilon^{th}$	Thermal strain
$q_{r}$	Heat radiation (W)	***C***	Material stiffness tensor (Pa)
$h_{c}$	Convective coefficient (${W/(m}^{2}K)$)	*E*	Young’s modulus (Pa)
*σ*	Stefan–Boltzmann constant (${W/(m}^{2}K^{4})$)	*ν*	Poisson’s ratio
*ε*	Radiation emissivity	*λ*	Plastic multiplier
*P*	Laser power (W)	*f*	Yield function (Pa)
*V*	Scanning velocity (mm/s)	*α*	Thermal expansion coefficient (1/K)
